# Supplementary material for: Inhibitory effect of capsule on natural transformation of Streptococcus pneumoniae
Source: mBio. 2025 Aug 28;16(10):e01394-25. doi: 10.1128/mbio.01394-25 (PMC12506006; doi:10.1128/mbio.01394-25)
Supplement: Supplemental figures — Figures S1 to S6. [file mbio.01394-25-s0001.pdf]

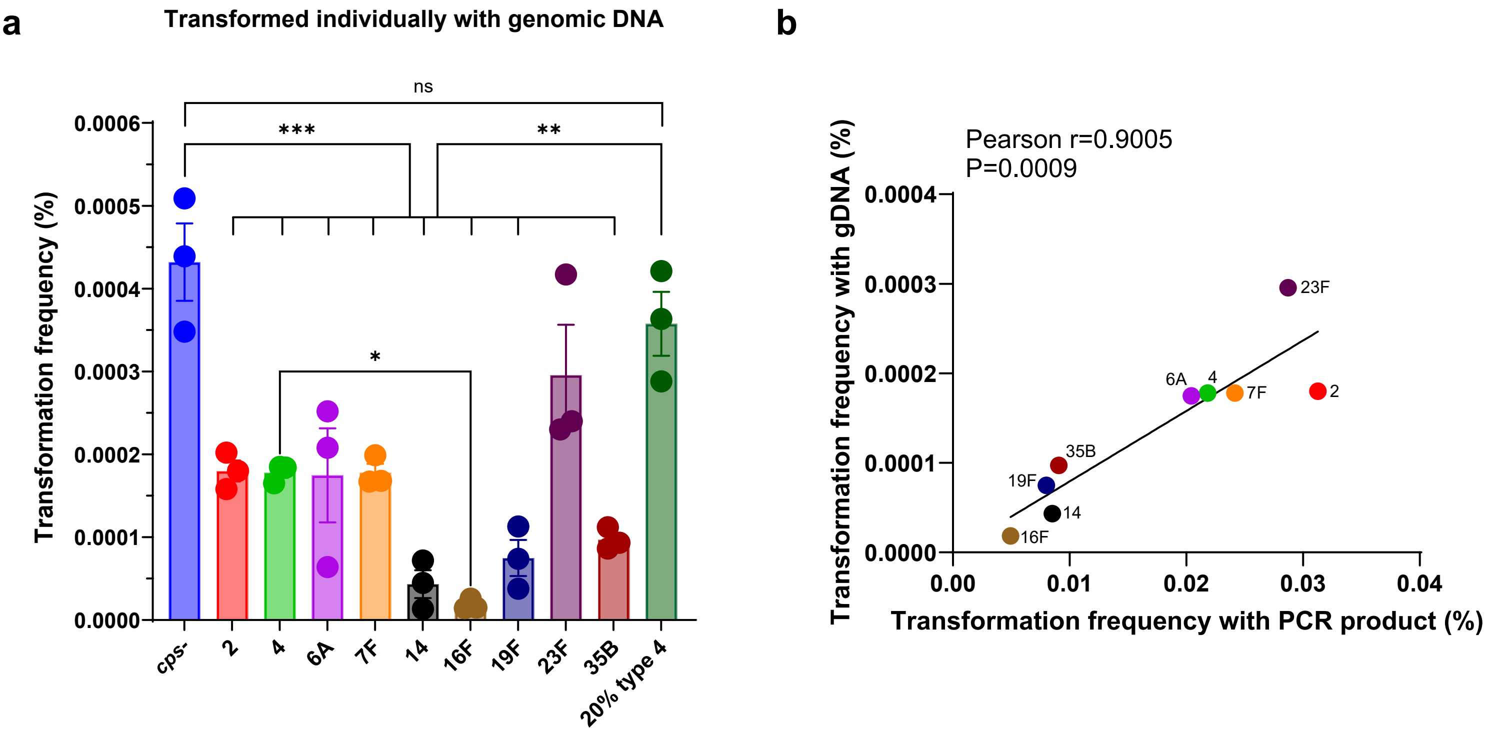
a
Transformed individually with genomic DNA
ns
***
**
*
Transformation frequency (%)
0.0006
0.0005
0.0004
0.0003
0.0002
0.0001
0.0000
cps-
2
4
6A
7F
14
16F
19F
23F
35B
20% type 4
b
Pearson r=0.9005
P=0.0009
Transformation frequency with gDNA (%)
0.0004
0.0003
0.0002
0.0001
0.0000
0.00
0.01
0.02
0.03
0.04
Transformation frequency with PCR product (%)
23F
6A
4
7F
2
35B
19F
14
16F

Fig S1. Transformation frequency of isogenic capsule-switch strains transformed with genomic DNA. (a) Transformation frequency of capsule-switch strains with 150 ng/mL CSP and genomic DNA from strain P2742 that bears a $Spec^R$ selection marker. Transformation frequency of the unencapsulated strain (*cps*-) or the strain with 20% type 4 capsule was compared to the rest strains using the ordinary one-way ANOVA with Šídák's multiple comparisons test. Transformation frequency of strains with different capsule types were compared using the ordinary one-way ANOVA with Tukey's multiple comparisons test. For brevity, only comparisons to *cps*-, type 4, or 20% type 4 were shown on the graph. (b) Correlation between transformation frequency of strains bearing different capsule types transformed with PCR product (Fig. 1b) and transformed with genomic DNA (Fig. S1a). Pearson correlation coefficients were computed.

a

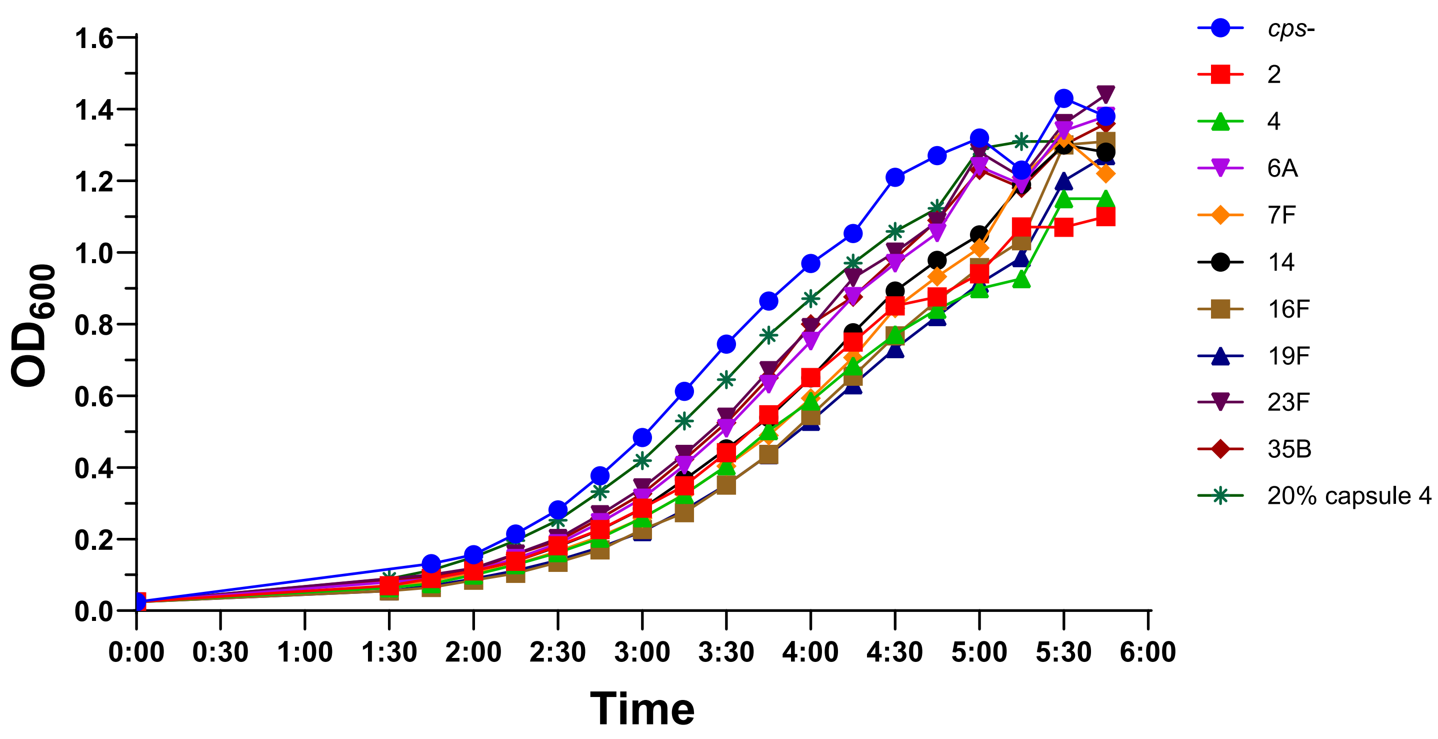

OD600
Time
0.0
0.2
0.4
0.6
0.8
1.0
1.2
1.4
1.6
0:00
0:30
1:00
1:30
2:00
2:30
3:00
3:30
4:00
4:30
5:00
5:30
6:00
cps-
2
4
6A
7F
14
16F
19F
23F
35B
20% capsule 4

b

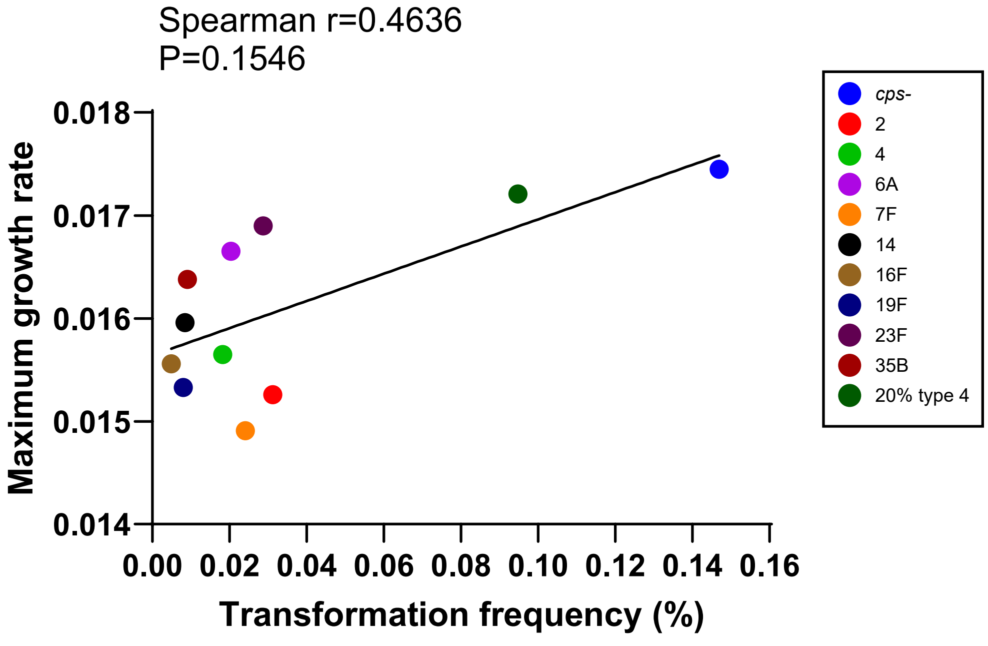

Correlation of transformation frequency with maximum growth rate
Spearman r=0.4636
P=0.1546
Maximum growth rate
Transformation frequency (%)
0.014
0.015
0.016
0.017
0.018
0.00
0.02
0.04
0.06
0.08
0.10
0.12
0.14
0.16
cps-
2
4
6A
7F
14
16F
19F
23F
35B
20% type 4

Fig S2. Growth rate of capsule-switch strains. (a) Growth curve of capsule-switch strains. Each strain was cultured in THY (pH=6.6) at 37°C until stationary phase. Optical density at 600 nm (OD_{600}) was measured every 15 min. (b) Correlation of transformation frequency with PCR product and maximum growth rate. The maximum growth rate was calculated as the slope of the log phase growth curve on a log scale. Spearman nonparametric correlation coefficients were computed. Strains are designated by their capsule type or amount for brevity.

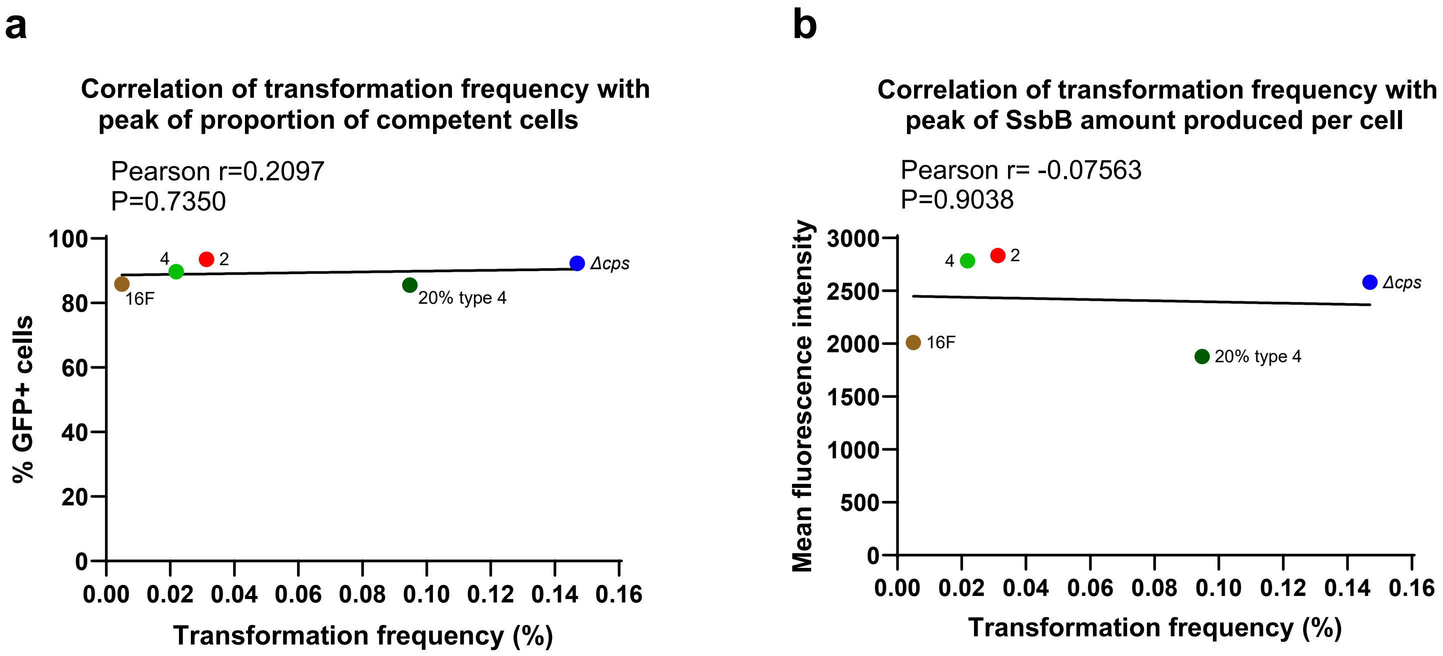
a
Correlation of transformation frequency with peak of proportion of competent cells
Pearson r=0.2097
P=0.7350
% GFP+ cells
Transformation frequency (%)
4
2
Δcps
16F
20% type 4
b
Correlation of transformation frequency with peak of SsbB amount produced per cell
Pearson r= -0.07563
P=0.9038
Mean fluorescence intensity
Transformation frequency (%)
4
2
Δcps
16F
20% type 4

Fig S3. Competence level of five selected representative strains examined by P$_{ssbB}$-*ssbB-gfp* reporters. (a) Correlation of transformation frequency with PCR product and the peak value of proportion of competent cells. Pearson correlation coefficients were computed. (b) Correlation of transformation frequency with PCR product and the peak value of amount of SsbB produced per cell. Pearson correlation coefficients were computed. Strains are designated by their capsule type or amount for brevity.

20% type 4

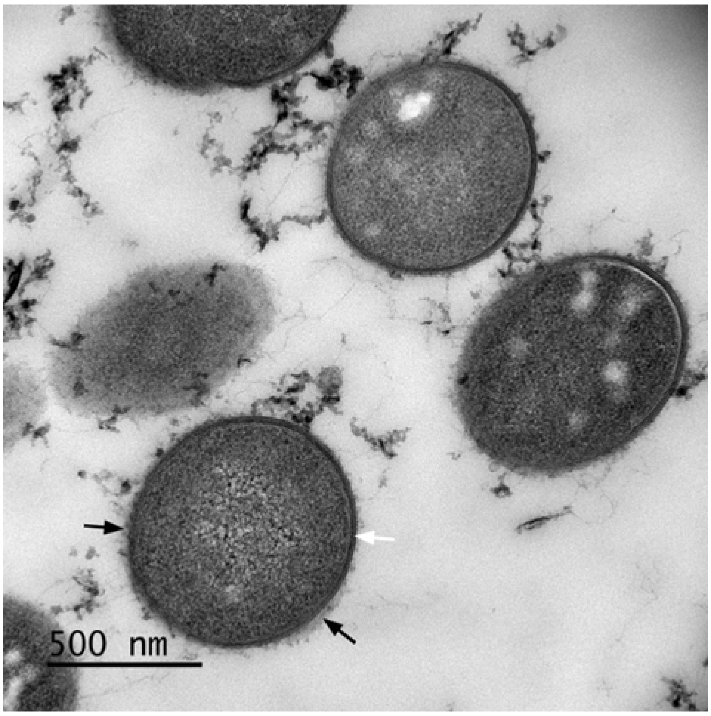

4

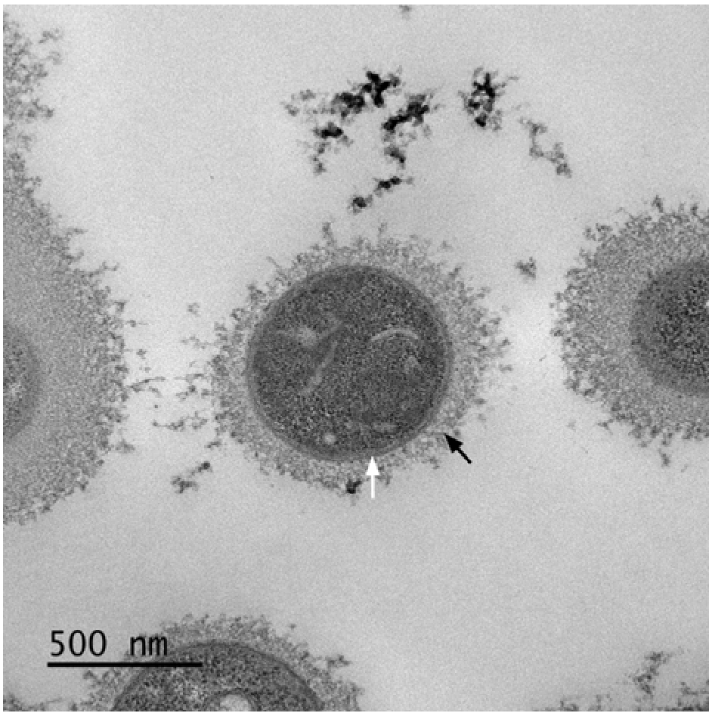

Fig S4. Transmission electron microscopy to visualize the capsule. Bacteria were chemically fixed with lysine acetate added, stained with 0.075% ruthenium red, and embedded in LR White. Black arrows indicate the capsule, and white arrows indicate the cell wall. Scale bar: 500 nm. Strains are designated by their capsule type or amount for brevity.

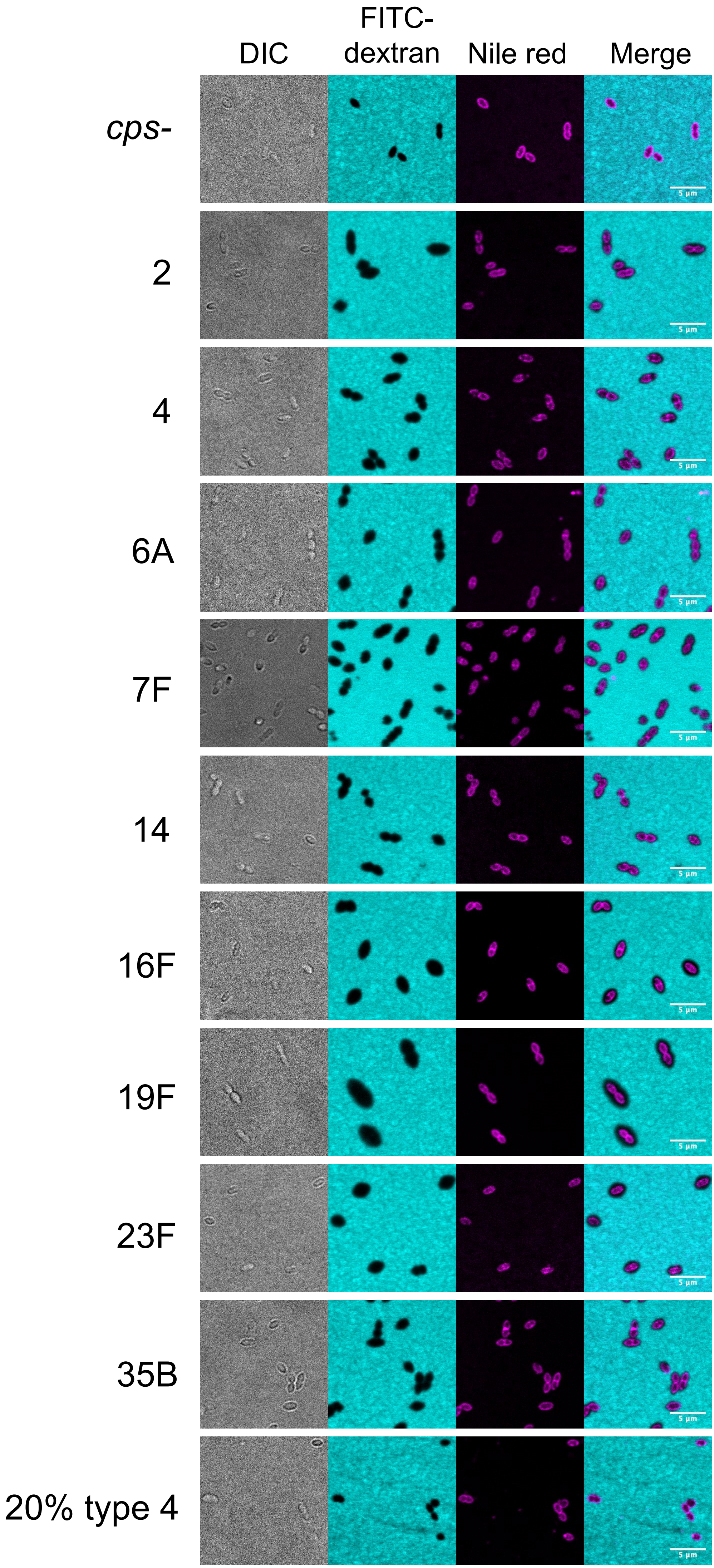

DIC
FITC-dextran
Nile red
Merge
cps-
2
4
6A
7F
14
16F
19F
23F
35B
20% type 4
5 μm

Fig S5. Capsule visualization via fluorescence light microscopy. Bacterial cell body including capsule was visualized as a shadow excluded from the background staining of FITC-conjugated dextran. The cell surface was visualized by staining membrane lipids with Nile red. Strains are designated by their capsule type or amount for brevity.

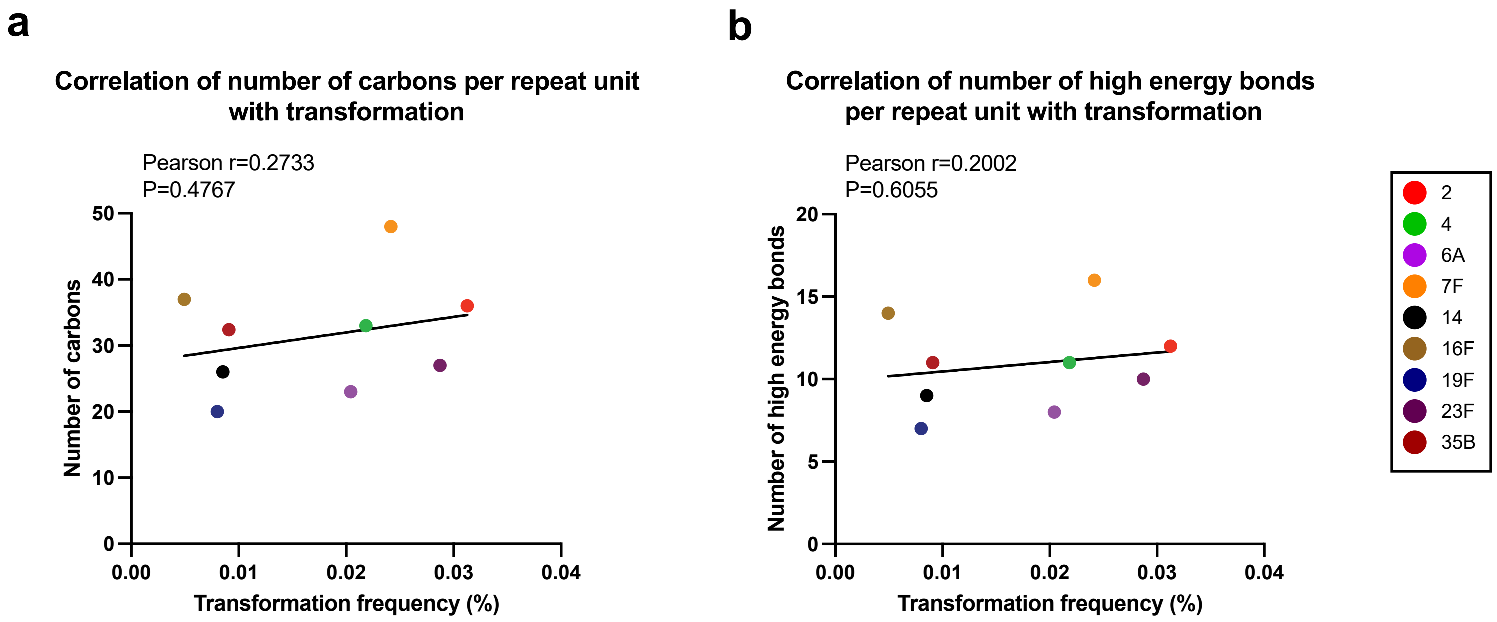
a
Correlation of number of carbons per repeat unit with transformation
Pearson r=0.2733
P=0.4767
Number of carbons
Transformation frequency (%)
b
Correlation of number of high energy bonds per repeat unit with transformation
Pearson r=0.2002
P=0.6055
Number of high energy bonds
Transformation frequency (%)
2
4
6A
7F
14
16F
19F
23F
35B

Fig S6. Correlation of transformation frequency with compositional metrics of capsular polysaccharides. (a) Correlation of transformation frequency with number of carbons per polysaccharide repeat unit. Pearson correlation coefficients were computed. (b) Correlation of transformation frequency with number of high energy bonds required to generate one polysaccharide repeat unit. Pearson correlation coefficients were computed. Compositional metrics data were obtained from the previous study (1). Strains are designated by their capsule type for brevity.

References

1. Weinberger DM, Trzcinski K, Lu YJ, Bogaert D, Brandes A, Galagan J, Anderson PW, Malley R, Lipsitch M. 2009. Pneumococcal capsular polysaccharide structure predicts serotype prevalence. PLoS Pathog 5:e1000476.
